# Clinical features and therapeutic outcomes of alveolar soft part sarcoma in children: A single-center, retrospective study

**DOI:** 10.3389/fonc.2022.1019911

**Published:** 2022-11-24

**Authors:** Zhichao Tan, Jiayong Liu, Ruifeng Xue, Zhengfu Fan, Chujie Bai, Shu Li, Tian Gao, Lu Zhang, Xinyu Wang

**Affiliations:** Key Laboratory of Carcinogenesis and Translational Research (Ministry of Education/Beijing), Department of Bone and Soft Tissue Tumor, Peking University Cancer Hospital and Institute, Beijing, China

**Keywords:** alveolar soft part sarcoma, pediatric, clinical features, targeted therapy, immunotherapy

## Abstract

**Background:**

Alveolar soft part sarcoma (ASPS) is a rare sarcoma that has been shown to be highly effective to antiangiogenic agents and immune checkpoint inhibitors, but most reported studies about ASPS were concentrated on adult population. In this study, we aimed to describe the clinical features and therapeutic outcomes of ASPS in children.

**Methods:**

We retrospectively reviewed the records of patients with ASPS in our institution since Jan 2015. All patients included in this study were pathologically confirmed ASPS and aged under 12 years at the time of initial diagnosis. Demographic characteristics, tumor sizes, primary tumor sites, metastasis, treatments used, therapeutic responses and survivals were evaluated.

**Results:**

We identified a total of 56 patients to be initially diagnosed as ASPS since Jan 2015. A predisposition of high occurrence in head and neck (32.1%) was observed (versus 41.1% in limbs and 21.4% in trunk). 26 (46.4%) patients developed metastasis at the time of diagnosis or during follow-up. Tumors in tongue, pharynx and larynx had the least likelihood to metastasize (7.7%, P<0.05). Observation was recommended for 15 stage IV patients with only pulmonary metastasis. 7 (46.7%) patients remained stable until last follow up. The 1-year PFS rate was 83.3% and median progression-free survival time (PFS) was 29.4 months. 15 patients with progressive disease received mono or combined therapy. 11 patients received PD-1 monotherapy. 2 patients achieved partial response and 5 stable disease. The overall response rate was 18.2%. The median PFS of this group was 22.0 months, and the 1-year PFS rate was 70.0%. 4 patients received a combination therapy of PD-1 inhibitors plus tyrosine kinase inhibitors. All of them remained stable. No disease-related death occurred during follow-up.

**Conclusions:**

ASPS exhibits a higher occurrence in head and neck in children. ASPS originating from glossopharyngeal region tends to have a lower metastasis rate. ASPS displays a more indolent growth pattern in children, which makes observation a preferable choice for children with sole pulmonary metastasis. Pediatric ASPS appears to be less effective to targeted therapy and immunotherapy than adults. The treatment of progressive ASPS in children remains challenging.

## Introduction

Alveolar soft part sarcoma (ASPS) is a rare histologic subtype of soft tissue sarcomas, which represents less than 1% of all soft tissue sarcomas (1). It is characterized by the frequent presence of the chromosomal rearrangement der (2)t(X;17)(p11;q25), leading to a chimeric APSCR1-TFE3 transcription factor (3). About one third of ASPS occur in children and adolescents, accounting for about 4.5% of all soft tissue sarcomas in this population (4). The majority of our knowledge regarding to ASPS comes from the adult population, and the size of the studies is relatively small. The largest study so far, which used the National Cancer Database, identified 293 ASPS patients aged 18 and over, and concluded that ASPS present with a high rate of metastasis (59%) and an indolent course when compared to other malignant soft tissue tumors (5). Resection remains the standard treatment when feasible. For stage IV ASPS, traditional cytotoxic chemotherapy enacted poor activity (overall response rate (ORR) less than 10%) (6). However, the recent progress in targeted and immune therapy had led to delighting results in ASPS. A recent meta-analysis evaluating the efficacy of immune checkpoint inhibitors in sarcomas reported an ORR of 35% in the ASPS subgroup (7). Tyrosine kinase inhibitors, such as cediranib and pazopanib, achieved an ORR of 20-35% (8, 9). Recently, a phase II study of TQB2450 (a PD-L1 antibody) in combination with anlotinib, achieved an ORR of 75.0% in the ASPS subgroup (10).

Pediatric series are very limited due to the extreme rarity of ASPS in children. The largest study consisted of 51 children and adolescents with ASPS from seven European Cooperative Groups (11). Another study, consisting of 69 patients, included patients younger than 30 years with ASPS from four major cancer centers in North America (12). So we conducted this retrospective, single-center study to better identify the clinical features and outcomes of ASPS in children,.

## Materials and methods

### Patient data

The clinical data of children (under 12 years old) diagnosed with ASPS since January, 2015 was retrospectively collected from our institution. The study was conducted in accordance with the Declaration of Helsinki. Informed consent was obtained for all patients. All patients included in this study were pathologically confirmed ASPS. Case records were analyzed for anatomical site of primary disease and metastasis, extent of surgery, adjuvant radiation, types and duration of medical therapy, response to therapy, time to progression or recurrence, and follow-up. The TNM postsurgical staging was employed.

### Therapeutic strategy

If possible, a wide excision of primary tumor would be recommended for all patients. For those with unresectable primary tumor or can be only resected with positive surgical margin (such as tumors located at orbit), radical radiotherapy would be conducted. After eradication of primary tumor, patients without metastasis and patients with only pulmonary metastasis were suggested to closely observe. We would start medical intervention for patients with extra-pulmonary metastasis, and those who were confirmed to have progressive disease during follow up. Tyrosine kinase inhibitors (TKIs) monotherapy, PD-1 inhibitors monotherapy, or a combination of these two agents would be employed according to the recommendation of the attending physician and the choice of the patient’s family. The PD-1 inhibitors we employed included pembrolizumab, torpalimab and sintilimab; the TKIs included anlotinib and pazopanib. We generally used 1/3-1/2 standard dose of these drugs, and the specific dose would be adjusted according to the age and weight of the patient. This therapeutic strategy was discussed and approved by the ethics committee of Peking University Cancer Hospital.

### Statistical analysis

Standard descriptive statistics were used. The means were compared by using independent sample t-test. Survival was analyzed using the Kaplan-Meier method. In the metastasis-free group, event-free survival (EFS) was defined as the time from the date of diagnosis to date of metastasize, relapse or last follow-up. In IV stage patients, for the observation group, progression-free survival (PFS) was defined as the time from the diagnosis of metastasis to the date of progression or last follow-up; for the treatment group, PFS was defined as the start of medical treatment to the date of progression or last follow-up. Overall survival (OS) was defined as time from the date of diagnosis to death or last follow-up. The monitor of primary tumor site and metastasis was conducted by using computed tomography (CT) or magnetic resonance imaging (MRI). For patients with measurable disease (tumor diameter greater or equal to 1cm), we used the Response Evaluation Criteria in Solid Tumors (RECIST, version 1.1) to evaluate the response to medical treatment. For patients without evaluable tumors (tumor diameter less than 1cm), we defined complete response (CR) as the all visible tumor disappeared with no residual disease, stable disease (SD) as tumor increased less than 5mm or decreased but still visible, progressive disease (PD) as the tumor increased more than 5mm. Significance (*P*<0.05, two-tailed) was determined by log-rank test with respect to EFS, PFS and OS, and by Cox regression models for the univariate and multivariate analysis for the following covariates: patient age, gender, primary tumor site, primary tumor size, medical treatment. Statistical analysis was completed using SPSS 25.0.

## Results

**Figure 1:**
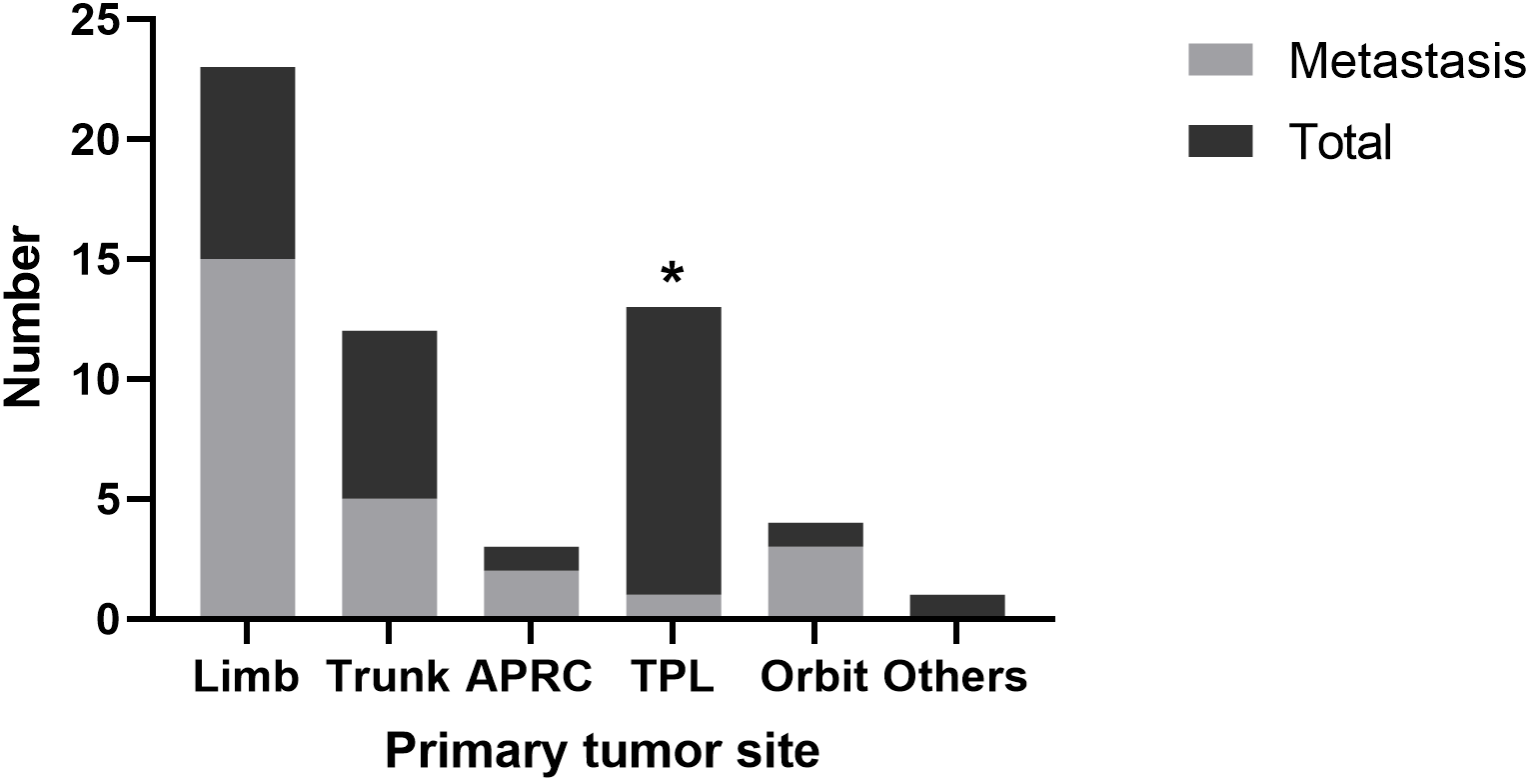
Tumor metastasis from different origin. APRC, abdominal, pelvic and retroperitoneal cavity; TPL, tongue, pharynx and larynx.

A total of 56 children under 12 years old were identified since 2015. The clinical characteristics of them are shown in **Table 1**. A predisposition of female dominance was observed (64.3%). The primary tumor was located mostly in limb (41.1%), head and neck (31.1%), and trunk (21.4%). Nearly half of the patients (46.4%) had metastasis at diagnosis or during follow-up. Different origins of tumor demonstrated different metastatic rate. Tumors located in tongue, pharynx and larynx were least likely to metastasize (7.7%, versus limb 65.2%, trunk 41.7%, abdominal and pelvic and retroperitoneal cavity, 66.7%, orbit 75%, *P*<0.05)(**Figure 1**). Interestingly, the diameter of tumor originating from this area (tongue, pharynx and larynx) was not significantly different from other parts (limb: 4.24cm, trunk: 3.42cm, abdominal and pelvic and retroperitoneal cavity: 4.6cm; orbit: 2.2cm; tongue, pharynx and larynx: 3.09cm, *P*>0.1). Gender and age were neither prognostic factor for metastasis.

**Table 1:** Characteristics of patients.

	Number (%)
Gender	
Male	20 (35.7)
Female	36(64.3)
Age (median, year)	6.5(1-12)
Primary tumor site	
Limb	23 (41.1)
Trunk	12 (21.4)
Abdominal, pelvic, retroperitoneal cavity	3 (5.4)
Head and neck	18 (32.1)
Tongue, pharynx and larynx	13 (23.2)
Orbit	4 (7.1)
others	1 (1.8)
Primary tumor diameter (median, cm)	3.6 (0.6-9.0)
T stage	
1	35 (62.5)
2	10 (17.9)
NA	11 (19.6)
N stage	
N0	54 (96.5)
N+	2 (3.5)
Metastasis	
No	30 (53.6)
Yes	26 (46.4)
Follow-up time (median, month)	34.7 (3.1-84.5)

NA, not available.

**Figure 2:**
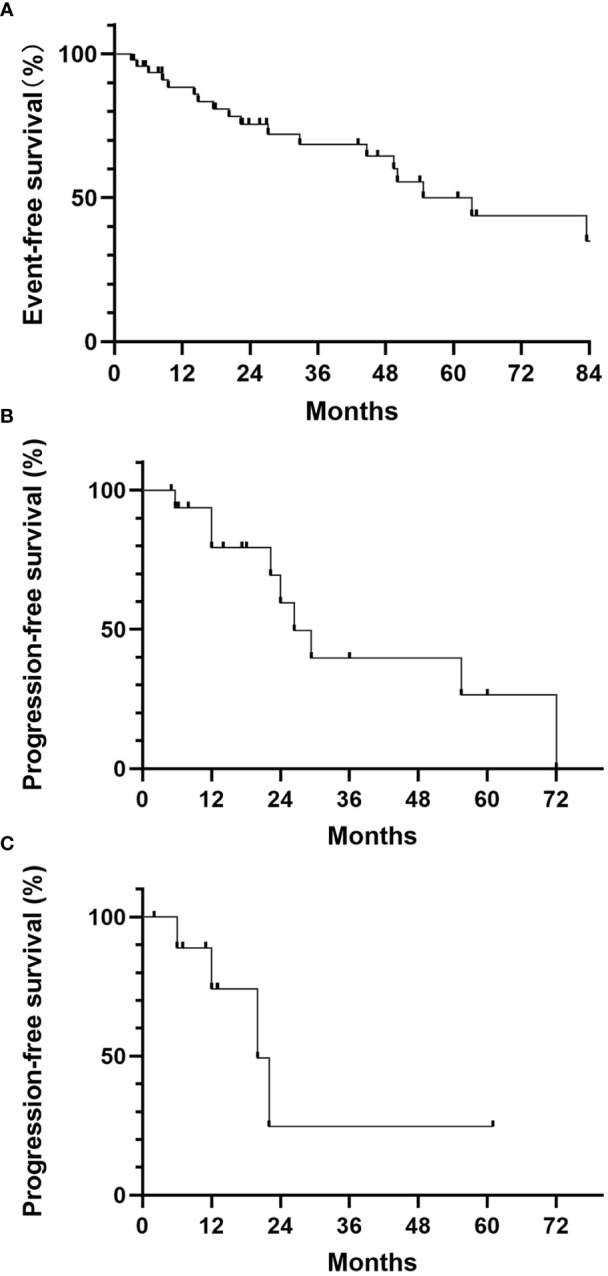
**(A)** Event-free survival of localized ASPS; **(B)** Progression-free survival for the observation group; **(C)** Progression-free survival for the PD-1 monotherapy group.

48 patients were metastasis-free when diagnosed, and 20 (41.7%) had metastasis during follow-up. The median event-free survival was 63.2 months (**Figure 2A**). 15 patients had only pulmonary metastasis, including those who were discovered when diagnosed or during follow-up. These patients were recommended to receive no medical treatment and observation. In this observation group, 7 patients (46.7%) remained stable during follow-up. The median PFS was 29.4 months, and the 1-year PFS rate was 83.3% (**Figure 2B**).

15 patients received medical treatment, including 9 patients with extra-pulmonary metastasis and 6 patients with progression of pulmonary metastasis during follow-up. No patient received TKIs monotherapy and 11 patients received PD-1 inhibitor monotherapy as initial treatment. 2 of 11 (18.2%) had PR, 6 had SD and 3 had PD. The median PFS for monotherapy group was 22.0 months, and the 1-year PFS rate was 70.0% (**Figure 2C**). 4 patients received TKIs and PD-1 inhibitors combination therapy. All of them remained SD. No CR were recorded in either monotherapy or combination therapy group. No disease-related death was reported during follow-up, thus the median OS was not reached.

## Discussion

As a rare cancer, it is hard to evaluate the biological behavior and therapeutic outcomes of ASPS with large-scale clinical trials, especially in pediatric population. Only several studies concentrated on pediatric and adolescent ASPS patients (11–13). However, there are some limitations in these studies. Firstly, both targeted therapy and immunotherapy have achieved good results in ASPS in recent years, and have changed the outcomes of many patients. Therefore, the results of these emerging therapies and the survival data of patients need to be updated. Secondly, these studies do not further distinguish between pediatric and adolescent populations. More data are needed to confirm the differences in clinical features, prognosis, and treatment response between ASPS in children and adolescents. Our study was aimed to evaluate clinical features of ASPS in the specific population: children under 12 years old. 56 patients were included in this study. As far as we know, this is the largest ASPS pediatric population reported to date.

We identified a relatively higher rate of occurrence and a lower rate of metastasis relating to a particular primary tumor site in head and neck, especially in glossopharyngeal region. In the adult ASPS population, tumors originating from head and neck only consisted of 3.4% (5); in the children and adolescent ASPS population, the ratio was 15%-19% (11, 12) while in our study it was 32.1%. On the contrary to its high occurrence, the metastatic rate of glossopharyngeal region was surprisingly low (7.7%). We are not the first to discover this phenomenon since Hagerty et al. also reported ASPS from head and neck had a lesser metastatic rate (40%) compared to extremity origins (73%) (5). Those researchers attributed it to the higher visibility of lesions arising from head and neck, which leads to an earlier medical intervention, but we doubt this theory because in our result, the diameter of tumors from this region was not significantly smaller than other origins, although the small sample size limited the validity. Tumor biology might be a contributing factor. Previous studies regarding head and neck sarcomas mostly reported distant metastatic rate around 20%. In 3 retrospective reviews examining a total of 403 patients with head and neck sarcomas, the authors reported a distant metastatic rate of 17.6%, with a total of 71 metastasis (14–16). Notably, Torosian et al. reported 565 patients with soft tissue sarcomas, in which 18% (52/237) of patients with extremity sarcomas and 19% (4/21) of patients with head and neck sarcomas had metastatic disease. This result was contradictory to the early intervention theory. We would like to suppose that head and neck should not be dealt as a whole when analyzing risk factors for metastasis. In our result, ASPS from head and neck had a metastatic rate of 23.5%, but tumors from glossopharyngeal region displayed a much lower distant metastatic rate compared to orbit origin (1/13 vs 3/4).

The 5-year survival rate of stage IV ASPS patients was approximately 50%-60% in recent research (5, 17). Thirty years ago, a same rate of 5-year survival rate was observed with localized disease (2). The data implies that the treatment has improved in recent years. But it still remains unclear when to start intervention with medical treatment because of its relatively indolent growth. In our result, about half of the patients with only pulmonary metastasis remained SD without any intervention, and the 1-year PFS rate reached up to 83.3%. We also noticed that there were some cases with only pulmonary metastasis remained a long period of stability during follow-up in the reports of other investigators (12). Moreover, there is further evidence that ASPS plays a more indolent course in young patients. Lieberman et al. reported that the frequency of metastasis at presentation increased with the age at diagnosis, and the median survival decreased with it: patients at age interval 0-9 years had 17% metastatic rate and median survival of 20 years, while patients at age over 30 years had 32% metastatic rate and median survival of only 5 years (2). Therefore, we would like to imply that observation can be the first choice for stage IV ASPS children with only pulmonary metastasis.

For patients with disease progression or extra-pulmonary metastasis, TKIs and/or PD-1 inhibitors were recommended by some authoritative institutions, such as the National Comprehensive Cancer Network (NCCN). In our study, no patient chose TKIs monotherapy and most patients chose PD1 inhibitors as the initial treatment, because they were concerned about the adverse effects and the rebound effects after drug resistance of TKIs. In adults, previous clinical trials demonstrated that PD-1 monotherapy achieved an ORR of 25%-37.2% (18, 19). However, only 16.7% of children with ASPS in our study responded to PD-1 inhibitors. 27.2% (3/11) patients had disease progression during monotherapy, which was higher compared to adult population (<10%) (18, 19). For combination therapy, all 4 patients who received TKIs and PD-1 inhibitors stayed SD. This was a disappointing result because in clinical trials recruiting adults, combination therapy achieved an ORR of 58.3%-75.0% (10, 20). These findings may imply that although ASPS in children is more indolent, but it is less sensitive to target and immune therapies. In the field of immunosenescence, there is a theory that tumors in the elderly have more mutations than the young, because of a long period of exposure to environment and/or intrinsic mutagens, which contributes more neoantigens to be targeted by T cells of the host immune system. This theory might be a potential explanation to our finding (21). But historic data about pediatric ASPS is very limited, and previous clinical trials excluded patients under 18 years old, therefore, we may need more clinical reviews to confirm the validity of this hypothesis.

There are some limitations in our study. First, due to the long-time span and technological reasons, not all patients had the testing of ASPL-TFE3 fusion gene. To make the pathologic confirmation, all histologic slides were reviewed by experienced pathologists and the necessary immunohistochemistry was performed, including TFE3. Second, selection bias was inevitable as a single center, retrospective study. We collected records of every confirmed ASPS patients to reduce the bias. Moreover, the imaging assessment was confirmed by both the clinician and the radiologist. Third, the number of patients receiving combination therapy was very limited, which undermines the validity of our results. Considering its rarity, further cooperation of large centers is essential to make high-level clinical evidence.

## Conclusion

ASPS affects head and neck more often in children. Head and neck should be more subdivided when analyzing risk factors, in which tumors originating from glossopharyngeal region had the lowest metastatic rate. The mechanism needs further exploration. ASPS displays a more indolent growth pattern in children than adults. For children with sole pulmonary metastasis, observation might be a preferrable choice. As for progressive disease, pediatric ASPS appears to be more resistant to both PD-1 inhibitors and TKIs than adults. Therefore, it is still challenging to find an effective treatment for children with ASPS.

## Data availability statement

The dataset included private data of patients. Requests to access the datasets should be directed to ZT, alveolarpku@126.com.

## Ethics statement

The studies involving human participants were reviewed and approved by the ethics committee of Peking University Cancer Hospital. Written informed consent to participate in this study was provided by the participants’ legal guardian/next of kin.

## Author contributions

ZT, JL, and RX contributed equally to this work. All authors contributed to the article and approved the submitted version.

## Conflict of interest

The authors declare that the research was conducted in the absence of any commercial or financial relationships that could be construed as a potential conflict of interest.

The reviewer LX declared a shared parent affiliation with the authors to the handling editor at the time of review.

## Publisher’s note

All claims expressed in this article are solely those of the authors and do not necessarily represent those of their affiliated organizations, or those of the publisher, the editors and the reviewers. Any product that may be evaluated in this article, or claim that may be made by its manufacturer, is not guaranteed or endorsed by the publisher.
